# Incorporation of second‐tier tests and secondary biomarkers to improve positive predictive value (PPV) rate in newborn metabolic screening program

**DOI:** 10.1002/jcla.24471

**Published:** 2022-05-02

**Authors:** Sarang Younesi, Bahareh Yazdani, Mohammad Mahdi Taheri Amin, Pourandokht Saadati, Soudabeh Jamali, Mohammad‐Hossein Modarresi, Shahram Savad, Saloomeh Amidi, Homayoun Razavi, Soudeh Ghafouri‐Fard

**Affiliations:** ^1^ Nilou Laboratory Tehran Iran; ^2^ 48439 Department of Medical Genetics School of Medicine Tehran University of Medical Sciences Tehran Iran; ^3^ 556492 Department of Medical Genetics Shahid Beheshti University of Medical Sciences Tehran Iran

**Keywords:** mass spectrometry, neonatal screening, second‐tier biomarkers

## Abstract

**Background:**

Nowadays, neonatal screening has become an essential part of routine newborn care in the world. This is a non‐invasive evaluation that evaluated inborn errors of metabolisms (IEMs) using tandem mass spectrometry (LC‐MS/MS) for the evaluation of the baby's risk of certain metabolic disorders.

**Methods:**

This retrospective study was conducted on 39987 Iranian newborns who were referred to Nilou Medical Laboratory, Tehran, Iran, for newborn screening programs of IEMs. We incorporated second‐tier tests and secondary biomarkers to improve positive predictive value (PPV).

**Results:**

Statistical data were recorded via call interviewing in 6–8 months after their screening tests. The overall prevalence of IEM was 1:975. The mean age of all participants was 3.9 ± 1.1 days; 5.1% of participants were over 13 days and 7.7% were preterm or underweight. A total of 11384 (29.4%) of the cases were born in a consanguineous family. The type of delivery was the cesarean section in 8332 (51.3%) valid cases. The neonatal screening results had an overall negative predictive value (NPV) of 100% and the overall PPV of 40.2%. The false‐positive rate was 0.15%.

**Conclusion:**

This study showed a high incidence of metabolic disease due to a high rate of consanguineous marriages in Iran and indicated that incorporation of second‐tier tests and secondary biomarkers improves PPV of neonatal screening programs.

## Graphical abstract

Herein, we showed newborn screening protocol to increase PPV and decrease recall.

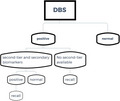

## 1 INTRODUCTION

The aim of newborn screening is to detect newborns with serious treatable disorders to facilitate appropriate interventions and avoid or ameliorate adverse outcomes. Mass biochemical testing of newborns was pioneered in the 1960s with the introduction of screening for phenylketonuria, a rare inborn error of metabolism, tested by using a dried blood spot sample. Most of these disorders are autosomal recessive. Inborn errors of metabolism (IEM) is a permanent and inherited biochemical disorder generally caused by lack of a functional enzyme, transmembrane transporter, or similar protein resulting in the blockage of the corresponding metabolic pathway. There may be an accumulation of metabolites before the metabolic block and/or deficiency in the ultimate products of the pathway. In metabolic diseases, an increase in one analyte in the metabolic pathway may result in an increase or a decrease in another analyte or production of secondary metabolic byproducts. Relevant ratios can be calculated and used to as secondary markers.^1^, ^2^

Detection of IEM through screening is the key element in the early treatment of these disorders. Undiagnosed and untreated disorders can cause irreversible mental retardation, physical disability, neurological damage, and even fatal outcomes.^3^

Tandem mass spectrometry (MS/MS) is a powerful technology that can be used for the rapid detection of many IEMs, including some diseases that could not be previously detected by other methods. MS/MS has some advantages over other currently available analytical methods and is widely used for newborn screening.^2^ MS/MS is mainly aimed at the detection of the urea cycle, amino acid, organic acid, and fatty acids abnormalities. The specificity and sensitivity of this method could be up to 99% for the mentioned disorders. This technique has been applied to measure over 50 metabolites; thus, it can screen more than 40 IEMs using dry blood samples.^4^

Each newborn screening laboratory must determine its own cut‐off values, normal values, and analytes ratios by measuring 1000–2000 normal dried blood spot (DBS) samples. The cut‐off value is set above 95% of the normal range, 10% lower than the SD cut‐off, and then, the analysis is repeated.^5^, ^6^

In a screening test, the positive predictive value (PPV) and negative predictive value (NPV) are informative parameters. Sensitivity and specificity should be provided for NPV and PPV calculation. PPV is the percentage of newborns with an abnormal result that have the disease. On the other hand, NPV is the percentage of cases with normal results that do not have this disease.

Second‐tier tests were developed to decrease false‐positive results and consequently increase PPV. Thus, it is essential to provide a separate testing protocol for second‐tier tests.^7^ These tests are available for some neonatal screening programs of IEMs, such as tyrosinemia type I, propionic acidemia, maple syrup urine disease (MSUD), congenital adrenal hyperplasia (CAH), hyperammonemia‐hyperornithinemia‐hyperhomocitrullinemia (HHH) syndrome, galactosemia, and lysosomal storage diseases.^8^, ^9^, ^10^, ^11^

This study is a retrospective investigation conducted on 39987 Iranian newborns referring to Nilou medical laboratory for the purpose of neonatal screening for IEMs. These babies were from 20 different provinces across Iran.

This study was performed to discover the current state of newborn screening in Iran and to evaluate the PPV, false‐positive rate (FPR), prevalence, detection rate (DR), and recall rate in this domain.

## 2 STUDY METHODS

In Nilou Medical Lab, MS/MS was applied to detect the IEMs, including amino acid disorders, fatty acids disorders, organic aciduria, and urea cycle disorders. Using this method, more than 50 metabolites were detected; thus, 40 IEMs were evaluated. All methods were carried out in accordance with relevant guidelines and regulations. All experimental protocols were approved by the institutional ethic committee of the Shahid Beheshti University of Medical Sciences. Informed consent was obtained from the parents.

## 3 CASES

In the present retrospective study, we collected the results of 39987 DBS samples sent for analysis to Nilou Medical Laboratory, Tehran, Iran. These samples were unique cases. The newborn screening test was performed on DBS samples between March 2017 and February 2020.

Most samples were taken on the 3rd‐7th days of birth. Each participant completed a questionnaire at the time of referral to the laboratory, containing clinical data, such as the birth date and weight, familial history, the presence of any metabolic disorders in siblings or other relatives, and type of feeding.

After taking heel stick blood of every participant, the relevant test was implemented to assess the 50 metabolic analytes, including amino acids and acyl carnitines using the instrument of Sciex Tandem Mass and Recipe ClinSpot^®^ LC‐MS/MS Complete Kit. In the cases of blood transfusion or after parenteral feeding, a second specimen was collected after seven days of birth. A second specimen was also collected after 15 days of birth in the cases of preterm or low birth weight babies (<2400 g) in cases suspicious for congenital hypothyroidism. Finally, collection of a third specimen was required after 30 days of birth for extremely low birth weight babies (<1800 g).

The cut‐off values used were initially set according to the literature. Subsequently, the data of 3000 healthy newborns were reviewed and the analyte concentrations corresponding to the 95^th^ percentile were calculated and considered as the reference levels. The cut‐off values were updated following every 6000 analytes assessments. We also applied age‐dependent criterion levels.^5^, ^6^

In this study, based on the determined cut‐offs, as the primary markers, we determined the secondary markers. For instance, in methylmalonic academia, an increase in propionyl carnitine (C3) level was considered as a primary biomarker^12^ and its ratio with acetylcarnitine (C2) was regarded as a secondary biomarker. Therefore, if the abnormal level was observed in this ratio, the probability of having the metabolic disease was increased. In these cases, a high level of secondary biomarkers without any recall was introduced for second‐tier or confirmatory tests.

Each sample with an abnormal result was repeated from the original DBS. If it was still high, the baby was recalled for confirming the diagnosis with follow‐up testing. We reduced the false‐positive rate and improved PPV using second‐tier tests (Table 1).

**TABLE 1:** Second‐tier tests and their evaluation methods

Analytes	Methods	Disorders	Reference
Allo‐isoleucine	HPLC	MSUD	^13^
Homocystein	LC‐MS/MS	Homocystinuria Maternal Vit B12 deficiency	^14^
Succinylacetone	LC‐MS/MS	Tyrosinemia type1	^15^

Since we did not detect any case of adenosylcobalamin synthesis defects (Cb1C, D), we did not include the relevant tests for this kind of disorder.

Because we used the RECIPE newborn screening, we could not detect succinylacetone levels. Thus, when we detected a high level of tyrosine in newborn screening, we evaluated succinylacetone levels using a PerkinElmer kit. Succinylacetone is considered a second‐tier test.

Finally, by entering the related information in the laboratory software (self‐developed) for reported disorders, the risk of IEMs was assessed and written answers, which included interpretive comments, were delivered to the families. Statistical data, such as baby status and metabolic symptoms, were recorded via telephone interviews 6–8 months after their screening tests.

## 4 RESULTS

After determining newborn screening cut‐off values in our laboratory, we observed that normal values of some acylcarnitines levels were lower than reference values from the literature. It can be due to different nutrition regimens. The newly established normal range caused a decrease in the false‐positive or false‐negative results. Detailed demographic and clinical data of cases are summarized in Table 2.

**TABLE 2:** Demographic and clinical data of patients (valid number column indicates the available data for each parameter)

Parameter	Valid No.	Median	Mean	SD	Minimum	Maximum
Maternal Age (Yrs.)	39987	31.7	32.3	4.7	18	51
In person/referral	39984		35188 (88.0%): In person 4796 (12.0%): Referral			
Gestational age at delivery	35473		38W+0D	1W+5D	26W+0D	41W+4D
Consanguinity	38721		11384 (29.4%): Related 27377 (70.6%): Non‐Related			
Type of delivery	16243		8332 (51.3%): Cesarean Section 7911 (48.7%): Normal Delivery			
Neonate age (Day)	39721	3.7	3.9	1.1	2 Days	13 Days
	2025 or 5.1% (>13 Days)				14 days	7 Years
Weight of neonates (Kg)	38546	2.97	3.17	0.44	0.92	4.5
Maternal problem at delivery	10837		116 (1.07%): had problems			
Neonatal problems	14967		3472 (23.2%): had problem			
Preterm Delivery	35473		1277 (3.6%): <37W+0D			
Under weight (<2.5 Kg)	38546		1580 (4.1%)			
Previous history of problems in sibling	9875		107 (1.09%): had problem			

The mean age of neonates was 3.9 ± 1.1 days; 5.1% of neonates were over 13 days and 7.7% were preterm or underweight. A total of 11384 cases (29.4%) came from a consanguineous marriage and the type of delivery in 8332 (51.3%) of valid cases was a cesarean section.

Detailed clinical problems of neonates in 3472 neonates from 14967 valid data are summarized in Table 3. A total of 3008 of cases had jaundice. Convulsion was reported in 344 (2.3%) cases. In addition, 164 (1.1%) of babies had no weight gain as the major problem. Finally, 254 (1.7%) of cases and 239 (1.6%) had hospitalization history and lethargy, respectively. At least 32.0% of patients had more than one problem in their histories.

**TABLE 3:** Detailed Clinical Problems of Neonates in 3472 Neonates (From 14967 valid data)

Parameter	No.	Percent (%)	Comments
No problems	11495	76.8	
Icterus	3008	20.1	
Convulsion	344	2.3	
No weight gain	164	1.1	
Vomiting	150	1.0	
Akathisia	45	0.3	
hospitalization	254	1.7	
Diarrhea	60	0.4	
Lethargy	239	1.6	
Bruising	59	0.4	
Respiratory problems	90	0.6	
Cerebral	15	0.1	
Blood exchange	75	0.5	
Eye problems	29	0.2	
Developmental disorders	46	0.3	
Total	4578		4578/3472= 1.32^a^

^a^ At least 32.0% of our symptomatic patients had more than 1 problem.

Prevalence, FPR, DR, and PPV of newborn screening by MS/MS are summarized in Table 4. PPV and FPR values for this disorder and its prevalence were 40%, 0.007%, and 1:19993, respectively. In addition, a total of 102 cases had abnormal results and finally, 41 of these cases were confirmed by second‐tier tests. Therefore, the overall PPV and FPR values and prevalence of these disorders were 40.2%, 0.15%, and 1:975, respectively.

**TABLE 4:** Prevalence, FPR, DR, and PPV of newborn screening by Tandem Mass Spectrometry in Nilou Laboratory

Disorders	Primary	Iformative ratios	2TT	Abnormal MS/MS		Confirmed by genetic tests	PPV	FN	Detection Rate	FPR	Prevalence	Others
				First sample	second sample							
Urea Cycle Disorders												
Arginemia	Arg	Arg/Orn Cit/Arg	–	3	3	1		–				
OTC^a^	Orn	–	–	2	2	1		–				
Total Urea Cycle Disorders				5	5	2	40.0 %	–	100%	0.007 %	1:19993	
Amino acid Disorders												
PKU	Phe	Phe/Tyr	–	32	24	12		–				
MSUD	Val‐Xle	Xle/Phe Xle/Ala Val/Phe	Allo‐Ile	23	9	8		–				
NKH^b^	Gly	Gly/Ala	–	7	7	2		–				
Tyrosinemia	Tyr	Tyr/Cit	Succiylaceton	72	14	3		–				
Total Amino acid Disorders				134	54	25	40.29%	–	100%	0.07%	1:1599	
Fatty acid Disorders												
MCAD^c^	C6 C8 C10	C8/C2 C8/C10	–	3	2	1		–				
CUD^d^	C0	Acs/Cit	–	8	6	1		–				
VLCAD^e^	C14 C14:1 C14:2 C16 C18 C18:1	C14:1/C12 C14:1/C2 C14:1/C4 C14:1/C16 C14:1/C5 C14:1/C8	–	4	4	1		–				
Total Fatty acid Disorders				15	12	3	25.0 %	–	100%	0.023 %	1:13329	
Organic acid Disorders												
MMA^f^	C3	C3/C2 C3/C16	HCY	8	5	4		–				
GA‐I^g^	C5DC	C5DC/C5OH C5DC/C8 C5DC/C16		9	6	5		–				
IVA^h^	C5	C5/C0 C5/C2 C5/C3		4	3	2		–				
Total Organic acid Disorders				21	14	11	52.4 %	–	100%	0.025	1:3635	
39987 DBS samples				175	92	41	44.60%	–	100%	0.13%	1:975	Recall = 0.23

^a^ Ornithine transcarbamylase deficiency (orotic aciduria).

^b^ Non Ketotic Hyperglycinemia.

^c^ Medium‐Chain Acyl‐CoA Dehydrogenase Deficiency.

^d^ Carnitine Uptake (Transport) Defect.

^e^ Very Long‐Chain Acyl‐CoA Dehydrogenase Deficiency.

^f^ Methylmalonic Acidemias; Glutaric Acidemia Type I.

^g^ Glutaric Acidemia Type I.

^h^ Isovalericacidemia (Isovaleryl CoA Dehydrogenase Deficiency, XLe (Leu, Ile, Allo‐Ile and Hydroxyproline), Acs (C0+C2+C3+C16+C18+C18:1).

After initial screening, 175 cases had positive results. We checked these screen‐positive results in the original DBS. If the repeated test on the first sample revealed positive results, available second‐tier tests were performed on the original samples. Positive second‐tier results were considered as a positive screen result and reported for clinicians. Using this strategy, we reached the final results for 83 newborns with confidence. For other 92 newborns, no second‐tier test was available, thus they were recalled for diagnostic tests. In 92 newborns with a first positive result, a new specimen was collected (Recall Rate =0.26), showing a 0.18% reduction in recall rate. For the second sample collected from the newborn, in the cases of amino acid disorders, plasma amino acids evaluation by the HPLC method was performed. For acylcarnitines abnormalities, plasma acylcarnitines, and urine organic acids were evaluated by LC‐MS/MS method. A positive second result was determined to be positive and was referred for further genetic testing. Finally, 41 infants were diagnosed with one of the IEMs.

As an example, propionyl carnitine level was shown to be elevated (5.9 µM) and its methionine level was 7 µM in the DBS sample of a 12 days newborn. We determined homocysteine (16 µM) level as a second‐tier test for vitamin B12 deficiency. This newborn was referred to another center for evaluation of folate, and Holo‐TC levels. These tests confirmed the diagnosis of maternal vitamin B12 deficiency.

According to this study data, phenylketonuria (classic) had more prevalence than other amino acid disorders. The highest FPR belonged to tyrosinemia (Type 1). In our laboratory, we tried to reduce FPR through performing second‐tier tests, such as succinylacetone evaluation and isovaleryl‐carnitine (C5) in DBS. C5 in MSUD patients was decreased dramatically.

Second‐tier tests were usually conducted on the first samples. However, when it was necessary, these tests were conducted on second samples obtained from newborns. In congenital hypothyroidism, in the cases of birth weight<2400 g or gestational age<36 weeks, when tyrosine levels were high, we recommended repeated test on the second samples even in the presence of normal succinylacetone. This has led to increase in the rate of re‐sampling in cases suspicious to tyrosinemia.

We additionally calculated the ratios of Xle/alanine/C5, and Val/alanine/C5. High Xle/Ala/C5 and Val/alanine/C5 ratios were the best indicators for MSUD.

In fatty acid disorders and organic acid disorder groups, medium‐chain acyl CoA dehydrogenase deficiency (MCAD) and glutaric acidemia type1 (GA) had the highest frequencies.

**FIGURE 1:**
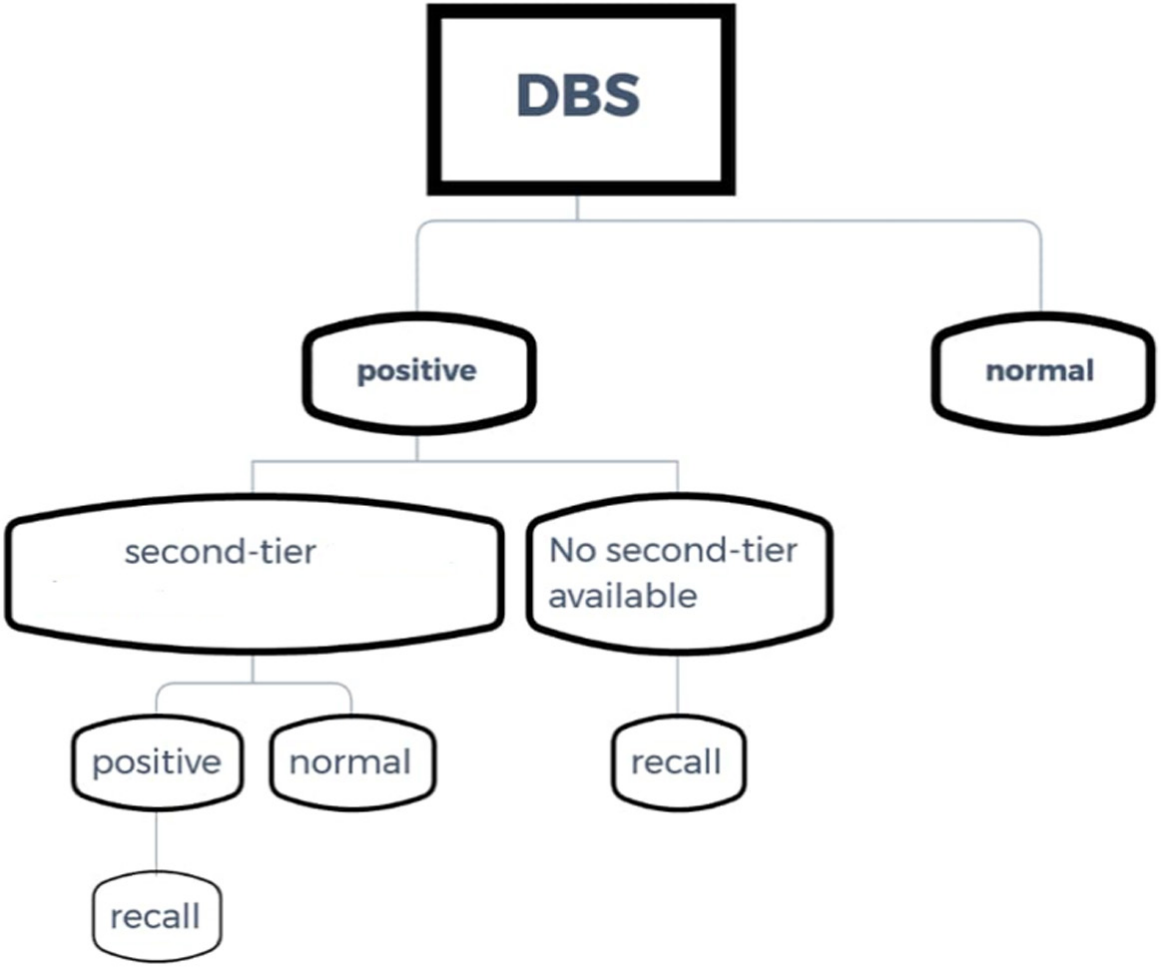
The newborn screening protocol to increase PPV and decrease recall

In this experience, we did not report any false‐negative results. Positive screen test results were confirmed by confirmatory and genetic tests. Figure 1 shows newborn screening protocol to increase PPV and decrease recall.

## 5 DISCUSSION

Newborn screening aims to diagnose inherited metabolic disorders before they cause irreversible sequels. Every newborn screening laboratory must determine its normal ranges and cut‐off values as well as secondary biomarkers ratio levels for enhancing the diagnostic value of related tests. The data of healthy newborns can be determined and the analyte concentrations corresponding to the 95^th^ percentile can be calculated and considered as reference levels for this purpose.^5^

McHugh et al. in 2011^16^ determined the cumulative percentiles of amino acids and acylcarnitines in DBS samples of approximately 25–30 million normal newborns from 130 sites in 45 countries. The obtained cumulative evidence has been used for the generation of 91 high and 23 low cut‐off target ranges.^16^ Our study is concordant with the study conducted by Wilcken et al. in 2008, Australia. They confirmed that adapting the cut‐off value is essential for newborn screening.^17^ The cut‐off values obtained from these studies were applied in the current study.

Our study showed that the overall PPV and FPR values and the prevalence of IEMs were 40.2%, 0.15%, and 1:975, respectively. These results are not concordant with those of Rinaldo et al.^18^ In the mentioned study on a total of 176,185 cases, the overall performance parameters were as follow: DR of 1:1,816, PPV of 37% (54% in 2006 till date), and FPR of 0.09%.^18^ The higher prevalence, PPV, and FPR in our study may be due to the high rate of consanguineous marriages in Iran. Moreover, since our laboratory is a referral laboratory, we collected samples from lots of high‐risk infants, for instance, those having positive screening results from other laboratories.

The prevalence of IMDs was as follows: 12 for PKU, 8 for MSUD, 5 for GA1, 4 for MMA, 3 for TYR, 2 for IVA, 2 for NKH, 1 for CUD, 1 for MCAD, 1 for VLCAD, and 2 for UCD. The prevalence of metabolic disease in our study was more than Tormis's study in Turkey, as described by Ozben.^11^ However, our results are consistent with the reports of the annual MENA (Middle East North Africa) region meeting, as the results of the conference showed a high rate of metabolic disease in infants in the MENA region due to the high endogamy and the high percentage of first‐cousin marriages.^19^ Our results are not concordant with the results of Kobarfard's study in Iran. In this study, the screening program was launched as a pilot study in six provinces of Iran in 2017 and a total of 145,169 babies were screened for the expanded panel of IMDs in two years. Positive samples were 3379 samples (2.3%) and 82 cases were confirmed as metabolic diseases (1 in 1770). The prevalence of IMDs was as follows: 1 for IVA, 22 for HPA/PKU, 7 for GA1, 2 for CUD, 2 for NKH, 12 for MSUD, 1 for GA2, 3 for PA, 6 for MMA, 4 for TYR, 4 for MCAD, 6 for UCD.^19^ Our results are comparable to those of Khneisser in Lebanon.^19^ Among the 276000 babies screened for the expanded MSMS panel, 19 cases with MSUD, six with CIT, three to five cases for each disease (MCAD, VLCAD, IVA, MMA, PA, GAI, and HMG), two with BKT, one with CPT‐I, and one with LCHAD were detected. The incidence of MCAD was lesser than what was anticipated, and relatively high consanguinity was detected in a cluster of 18 of 19 MSUD cases that had been detected in a geographically isolated region of 40,000 residents.^19^

The prevalence of metabolic disorders in our study was more than in European countries and the United States. It might be a consequence of the high rate of consanguinity and endogamy and the relatively large family size in Iran. Therefore, we observed a high prevalence of autosomal recessive conditions and other monogenic disorders in this population.

Easy second‐tier tests are now available to decrease the FPR, improve the PPV, and reduce false tests.^20^, ^21^ Recall rates in various programs in different parts of the world range from 0.01% to 13.3%; the difference being mainly due to various screening methodologies, including screening protocols, different laboratory techniques, and kits, site of sample collection, and different recall criteria (cut‐offs) and second‐tier tests.^4^, ^18^, ^22^ This study showed that through establishing our own cut‐off values, using secondary biomarkers, and application of second‐tier tests, we can reduce recall and false‐positive results (about 0.18%). The rate of recall in this study was 0.26%. Therefore, it causes early diagnosis and lifesaving treatment to infants with hereditary metabolic diseases. As a result, the FPR was decreased and the PPV was increased.

Similar findings were reported by Ombrone et al. in Italy in 2016.^2^ They proved that the new challenge for the future will be reducing the FPR by using second‐tier tests, avoiding false‐negative results by using new specific biomarkers and introducing new treatable disorders in newborn screening programs.^2^ Finally, we could identify three cases with MSUD using the secondary biomarker by calculating the ratio of Xle/alanine/C5 on the same primary sample, all of which show an increase in alloisoleucine level.

It is worth mentioning that levels of amino acids and acylcarnitines are different in the first and second weeks. Similarly, levels of secondary biomarkers are different. Thus, in order to avoid false‐negative or false‐positive results, we used age‐dependent cut‐off values.

In newborn screening, for improving the PPV and decreasing the false‐positive and recall rates, it is important to develop a second‐tier test, select conservative cut‐off, and secondary biomarkers points. Moreover, applying age‐dependent criteria for cut‐off levels can be helpful. All these strategies markedly enhance the sensitivity and specificity of the initial test in the newborn screening program.

## AUTHOR CONTRIBUTIONS

SGF and SY wrote the draft and revised it. MHM and SS designed and supervised the study. BY and MMTA analyzed the data. PS, SJ, SA and HR performed the experiment and collected the data. All the authors read and approved the submitted version.

## CONFLICT OF INTEREST

The authors declare they have no conflict of interest.

### ETHICAL APPROVAL

All procedures performed in studies involving human participants were in accordance with the ethical standards of the institutional and/or national research committee and with the 1964 Helsinki Declaration and its later amendments or comparable ethical standards. Informed consent forms were obtained from all study participants. The study protocol was approved by the ethical committee of Shahid Beheshti University of Medical Sciences. All methods were performed in accordance with the relevant guidelines and regulations.

### CONSENT OF PUBLICATION

Not applicable.

## Data Availability

The analyzed data sets generated during the study are available from the corresponding author on reasonable request.
